# Differential role of RIP1 in Smac mimetic-mediated chemosensitization of neuroblastoma cells

**DOI:** 10.18632/oncotarget.6308

**Published:** 2015-11-12

**Authors:** Sebastian Czaplinski, Behnaz Ahangarian Abhari, Alica Torkov, Dominik SeggewiΔ, Manuela Hugle, Simone Fulda

**Affiliations:** ^1^ Institute for Experimental Cancer Research in Pediatrics, Goethe-University, Frankfurt, Germany; ^2^ German Cancer Consortium (DKTK), Heidelberg, Germany; ^3^ German Cancer Research Center (DKFZ), Heidelberg, Germany

**Keywords:** Smac mimetic, apoptosis, RIP1, BCL-2 proteins, neuroblastoma

## Abstract

We explored the potential of Smac mimetics, which antagonize Inhibitor of Apoptosis (IAP) proteins, for chemosensitization of neuroblastoma (NB). Here, we report that Smac mimetics, e.g. BV6, prime NB cells for chemotherapeutics including the topoisomerase II inhibitor doxorubicin (DOX) and vinca alkaloids such as Vincristine (VCR), Vinblastine (VBL) and Vinorelbine (VNR). Additionally, BV6 acts in concert with DOX or VCR to suppress long-term clonogenic growth. While BV6 causes rapid downregulation of cellular IAP (cIAP)1 protein and nuclear factor-kappaB (NF-κB) activation, DOX/BV6- or VCR/BV6-induced apoptosis occurs independently of NF-κB or TNFα signaling, since overexpression of dominant-negative IκBα superrepressor or the Tumor Necrosis Factor (TNF)α-blocking antibody Enbrel fail to block cell death. Mechanistic studies reveal that Receptor-interacting protein (RIP)1 is required for DOX/BV6-, but not for VCR/BV6-induced apoptosis, since transient or stable knockdown of RIP1 or the pharmacological RIP1 inhibitor necrostatin-1 significantly reduce apoptosis. By comparison, VCR/BV6-mediated apoptosis critically depends on the mitochondrial pathway. VCR/BV6 cotreatment causes phosphorylation of BCL-2 during mitotic arrest, enhanced activation of BAX and BAK and loss of mitochondrial membrane potential (MMP). Additionally, overexpression of BCL-2 profoundly suppresses VCR/BV6-induced apoptosis. Thus, BV6 sensitizes NB cells to chemotherapy-induced apoptosis via distinct initial signaling mechanisms depending on the chemotherapeutic drug. These findings provide novel mechanistic insights into Smac mimetic-mediated chemosensitization of NB.

## INTRODUCTION

NB is a common solid tumor in childhood and patients with advanced or relapsed disease still harbor a poor prognosis [1]. Hence, there is a high medical need to develop novel therapeutic strategies. The efficacy of chemotherapy, one of the key pillars of current treatment protocols for NB, relies on functional cell death programs in tumor cells [2]. Therefore, strategies that embark on activation of programmed cell death may open new perspectives to enhance the chemosensitivity of cancer cells [3].

There are two principal pathways of apoptosis that involve activation of caspases as effector molecules, i.e. the extrinsic (i.e. death receptor) pathway and the intrinsic (i.e. mitochondrial) pathway [2]. The extrinsic pathway is activated by binding of ligands to death receptors of the TNF receptor superfamily on the cell's surface, for example Tumor-Necrosis-Factor-related apoptosis-inducing ligand (TRAIL) to TRAIL receptors or TNFα to TNF receptors [4]. The intrinsic pathway relies on the release of mitochondrial proteins such as cytochrome *c* and second mitochondria-derived activator of caspases (Smac) into the cytosol where cytochrome *c* mediates caspase activation while Smac antagonize IAP proteins [5].

Cell death pathways are tightly regulated by pro- and antiapoptotic proteins. The BCL-2 family of proteins plays an important role in the control of mitochondrial outer membrane permeabilization and comprises antiapoptotic members such as BCL-2, BCL-X_L_ and MCL-1 and proapoptotic members such as BAX and BAK [5]. Within the IAP family of proteins, x-linked IAP (XIAP), cIAP1 and cIAP2 are key regulators of programmed cell death [6]. While XIAP inhibits caspase activation by binding to caspase-3, -7 and -9, cIAP proteins are involved in the regulation of canonical and non-canonical NF-κB signaling, e.g. by their ability to promote ubiquitylation of RIP1 [6].

The targeting of IAP proteins has gained substantial attention over the last years, as elevated expression of IAP proteins is commonly found in many cancer types [6]. Small-molecule IAP antagonists that mimick the IAP-binding motif of Smac, i.e. Smac mimetics, have been developed and shown to elicit cell death in various cancers either alone or in combination therapies [6].

We previously reported that inhibition of IAP proteins sensitizes NB cells for TRAIL- or γ-irradiation-induced apoptosis [7, 8]. Recent evidence suggests that IAP inhibition by Smac mimetic may also provide a mean to increase chemosensitivity of NB cells; however, the underlying mechanisms have so far remained elusive [9]. Therefore, the aim of our study was to investigate the ability of Smac mimetics to sensitize NB cells to chemotherapy and to identify the underlying molecular mechanisms of action.

## RESULTS

### Smac mimetics synergize with DOX and vinca alkaloids to induce apoptosis in NB cells

**Figure 1:**
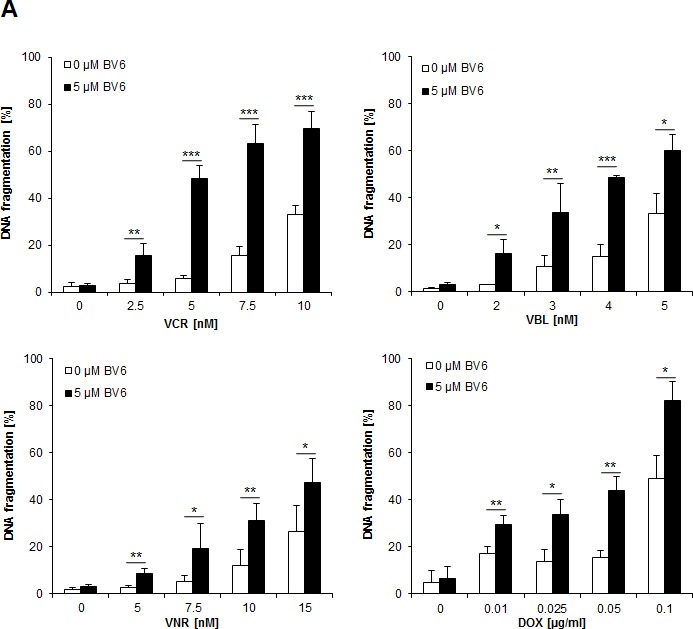

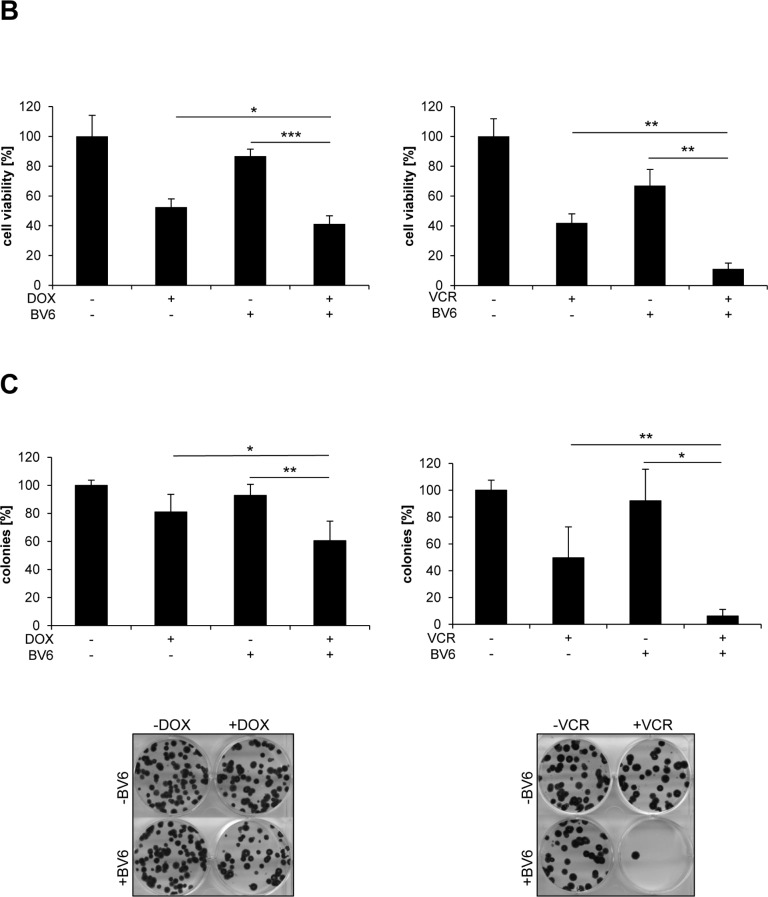
BV6 synergizes with DOX and vinca alkaloids to induce apoptosis in NB cells **A.** SH-EP cells were treated with indicated concentrations of DOX, VCR, VBL, VNR and/or 5 μM BV6 for 72 hours. Apoptosis was determined by analysis of DNA fragmentation of PI-stained nuclei using flow cytometry. Data are shown as mean and SD of three independent experiments performed in triplicate; *, *P* < 0.05; **, *P* < 0.01; ***, *P* < 0.001. **B.** SH-EP cells were treated with either 5 μM BV6 and/or 0.05 μg/ml DOX or 5 μM BV6 and/or 5 nM VCR for 48 hours. Cell viability was measured by crystal violet assay and is expressed as a percentage of untreated cells. Data are shown as mean and SD of three independent experiments performed in triplicate; *, *P* < 0.05; **, *P* < 0.01; ***, *P* < 0.001. **C.** SH-EP cells were treated with either 5 μM BV6 for 11 hours and/or 0.05 μg/ml DOX for 1 hour or 5 μM BV6 and/or 5 nM VCR for 72 hours. Colony formation was assessed as described in Material and Methods. The number of colonies is expressed as percentage of controls (upper panels) and representative images are shown (lower panels). Data are shown as mean and SD of three independent experiments performed in triplicate; *, *P* < 0.05; **, *P* < 0.01.

To investigate chemosensitization of NB cells by Smac mimetics, we tested the bivalent Smac mimetic BV6 in combination with subtoxic doses of vinca alkaloids or the topoisomerase II inhibitor DOX, which are commonly used in clinical protocols for the treatment of NB. We used the NB cell line SH-EP, which was previously shown to represent a suitable *in vitro* model of NB and to express key apoptosis regulators such as caspase-8 [10, 11]. Importantly, we found that BV6 cooperated with several vinca alkaloids, including VCR, VBL and VNR, as well as with DOX to significantly increase DNA fragmentation, which was used as a characteristic parameter to determine apoptosis (Figure 1A). Calculation of combination index (CI) revealed that BV6 acted in a synergistic manner together with DOX or VCR to induce apoptosis (suppl. Tab. 1). We confirmed the cooperative drug interactions by employing crystal violet assay as another method to determine cytotoxicity. BV6 acted in concert with DOX or VCR to significantly reduce cell viability compared to treatment with DOX or VCR alone (Figure 1B). Also, we extended our study to additional NB cell lines and to another Smac mimetic. Similarly, BV6 significantly enhanced VCR-mediated apoptosis in other NB cell lines (suppl. Figure 1A), and a pharmacologically distinct Smac mimetic (i.e. IAP inh. 3) significantly increased VCR- and DOX-induced apoptosis (suppl. Figure 1B). Furthermore, we asked whether the combination treatment affects long-term clonogenic survival of NB cells. Indeed, BV6 cooperated with DOX or VCR to significantly suppress colony formation compared to treatment with either agent alone (Figure 1C, suppl. Figure 1C). In contrast to NB cells, BV6 did not enhance the cytotoxicity of DOX or VCR against non-malignant peripheral blood lymphocytes (PBLs), pointing to some tumor selectivity (suppl. Figure 1D). To explore whether BV6 also sensitizes chemoresistant cancer cells, we used a rhabdomyosarcoma model of VCR resistance. VCR-resistant cells were refractory to BV6/VCR cotreatment (suppl. Figure 1E), indicating that the combination does not bypass VCR resistance.

Together, these experiments show that Smac mimetics cooperate with subtoxic doses of DOX or vinca alkaloids to induce apoptosis and to suppress long-term clonogenic survival of NB cells.

### BV6 cooperates with chemotherapeutics to trigger caspase activation and caspase-dependent apoptosis

**Figure 2:**
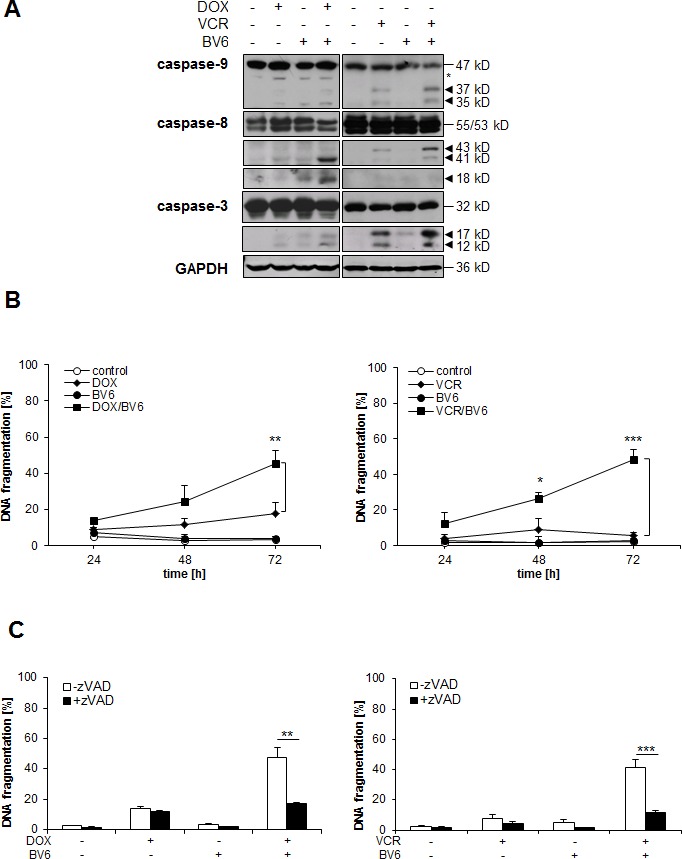
BV6 cooperates with chemotherapeutics to trigger caspase activation and caspase-dependent apoptosis **A.** SH-EP cells were treated with either 5 μM BV6 and/or 0.05 μg/ml DOX for 24 hours or 5 μM BV6 and/or 5 nM VCR for 18 hours. Activation of caspase-9, caspase-8 and caspase-3 was analyzed by Western blotting, arrowheads indicate active cleavage fragments, asterisk denotes an unspecified band, expression of GAPDH served as loading control. **B.** SH-EP cells were treated with either 5 μM BV6 and/or 0.05 μg/ml DOX or 5 μM BV6 and/or 5 nM VCR for indicated times. Apoptosis was determined by analysis of DNA fragmentation of PI-stained nuclei using flow cytometry. Data are shown as mean and SD of three independent experiments performed in triplicate; *, *P* < 0.05; **, *P* < 0.01; ***, *P* < 0.001. **C.** SH-EP cells were treated with either 5 μM BV6 and/or 0.05 μg/ml DOX or 5 μM BV6 and/or 5 nM VCR in the presence or absence of 20 μM zVAD.fmk for 72 hours. Apoptosis was determined by analysis of DNA fragmentation of PI-stained nuclei using flow cytometry. Data are shown as mean and SD of three independent experiments performed in triplicate; **, *P* < 0.01; ***, *P* < 0.001.

To better understand the molecular mechanisms underlying the cooperative induction of apoptosis that we identified for BV6 together with chemotherapeutics, we monitored activation of caspases by Western blotting. BV6 acted in concert with DOX or VCR to trigger cleavage of caspase-8 and caspase-3 into active fragments (Figure 2A). Cleavage of caspase-9 into active p37/p35 fragments was in particular observed upon cotreatment with VCR/BV6 (Figure 2A). Kinetic analysis of apoptosis revealed that this proteolytic activation of the caspase cascade occurred prior to the increase in apoptosis (Figure 2B), suggesting that activation of the caspase cascade contributes to apoptosis. To determine whether caspase activation is indeed required for apoptosis, we tested the effects of the pan-caspase inhibitor zVAD.fmk. Importantly, addition of zVAD.fmk significantly reduced DOX/BV6- or VCR/BV6-induced caspase activation and apoptosis (Figure 2C, suppl. Figure 1F). These findings show that BV6 cooperates with chemotherapeutics to trigger caspase activation and caspase-dependent apoptosis.

### NF-κB and TNFα signaling are dispensable for DOX/BV6-or VCR/BV6-induced apoptosis

**Figure 3:**
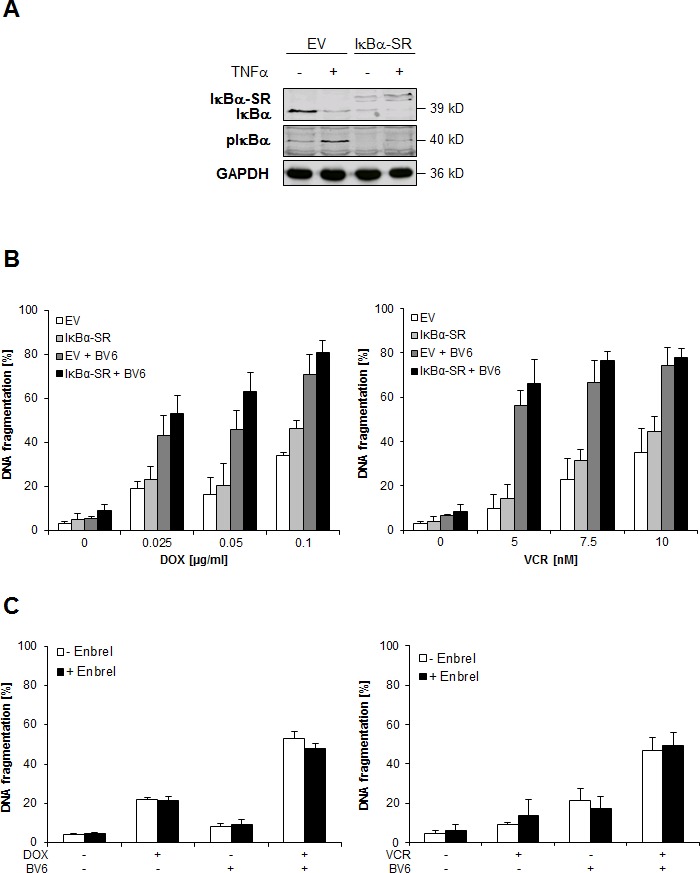
NF-κB and TNFα signaling are dispensable for DOX/BV6 or VCR/BV6-induced apoptosis **A** and **B.** SH-EP cells were stably transduced with empty vector (EV) or IκBα superrepressor (IκBα-SR). Cells were treated with 10 ng/ml TNFα for 1 hour and expression of IκBα, IκBα-SR and phospho-IκBα was assessed by Western blotting (A). SH-EP cells were treated with 5 μM BV6 and indicated concentrations of DOX or VCR for 72 hours (B). Apoptosis was determined by analysis of DNA fragmentation of PI-stained nuclei using flow cytometry. Data are shown as mean and SD of three independent experiments performed in triplicate. **C.** SH-EP cells were treated with either 5 μM BV6 and/or 0.05 μg/ml DOX or 5 μM BV6 and/or 5 nM VCR in the presence of absence of 50 μg/ml Enbrel for 72 hours. Apoptosis was determined by analysis of DNA fragmentation of PI-stained nuclei using flow cytometry. Data are shown as mean and SD of three independent experiments performed in triplicate.

Smac mimetics such as BV6 have been shown to trigger NF-κB activation and autocrine/paracrine TNFα signaling upon depletion of cIAP proteins [12]. We confirmed that treatment with BV6 resulted in rapid downregulation of cIAP1 protein (suppl. Figure 2A). In order to determine the role of NF-κB in DOX/BV6-or VCR/BV6-induced apoptosis, we overexpressed dominant-negative IκBα-SR to abolish NF-κB signaling (Figure 3A). We previously demonstrated that overexpression of IκBα-SR blocks activation of both canonical and non-canonical NF-κB pathways upon treatment with BV6 [13]. Control experiments confirmed that IκBα-SR overexpression inhibited TNFα-stimulated phosphorylation of IκBα, used as a marker for NF-κB activation (Figure 3A), and significantly enhanced TNFα-induced apoptosis and loss of cell viability (suppl. Figure 2B, 2C). However, inhibition of NF-κB by IκBα-SR failed to protect cells from DOX/BV6-or VCR/BV6-induced apoptosis (Figure 3B). To test the involvement of an autocrine/paracrine TNFα loop, we used the TNFα-blocking antibody Enbrel. Enbrel failed to rescue cells from caspase activation and apoptosis upon treatment with BV6 together with DOX or VCR (Figure 3C, suppl. Figure 1F). As positive control, we used MDA-MB-231 breast carcinoma cells, as we previously reported that they undergo apoptosis upon treatment with BV6 in a TNFα-dependent manner [13]. Addition of Enbrel profoundly blocked TNFα-induced apoptosis in MDA-MB-231 cells (suppl. Figure 2D). This set of data demonstrates that NF-κB and TNFα signaling are dispensable for DOX/BV6-or VCR/BV6-induced apoptosis.

### RIP1 is required for DOX/BV6-induced apoptosis, but dispensable for VCR/BV6-mediated apoptosis

**Figure 4:**
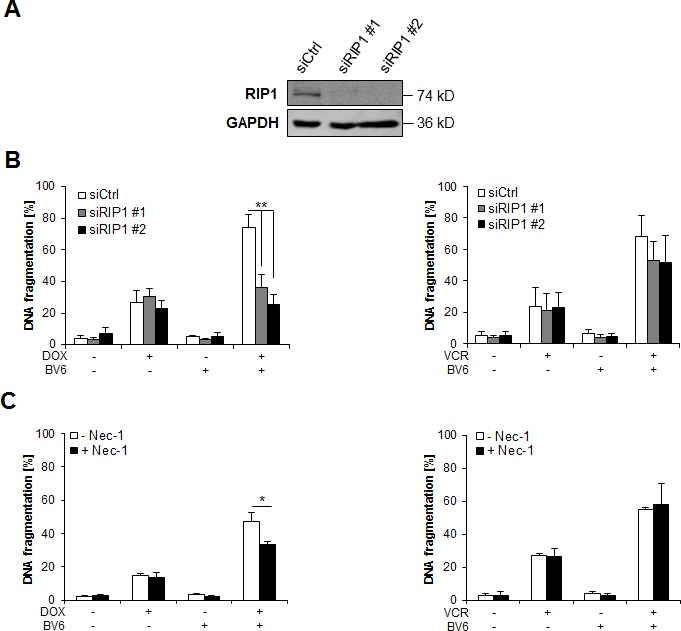
RIP1 is required for DOX/BV6-induced apoptosis, but dispensable for VCR/BV6-mediated apoptosis **A** and **B.** SH-EP cells were transiently transfected with non-silencing siRNA (siCtrl) or two different constructs targeting RIP1 (siRIP1 #1, siRIP1 #2) and expression of RIP1 was analyzed by Western blotting; GAPDH served as loading control (A). SH-EP cells were treated with either 5 μM BV6 and/or 0.05 μg/ml DOX or 5 μM BV6 and/or 5 nM VCR for 72 hours (B). Apoptosis was determined by analysis of DNA fragmentation of PI-stained nuclei using flow cytometry. Data are shown as mean and SD of three independent experiments performed in triplicate; **, *P* < 0.01. **C.** SH-EP cells were treated with either 5 μM BV6 and/or 0.05 μg/ml DOX or 5 μM BV6 and/or 5 nM VCR for 72 hours in the presence of absence of 30 μM Nec-1. Apoptosis was determined by analysis of DNA fragmentation of PI-stained nuclei using flow cytometry. Data are shown as mean and SD of three independent experiments performed in triplicate; *, *P* < 0.05.

Since we previously reported that RIP1 is required for Smac mimetic-mediated sensitization for TRAIL-induced apoptosis of NB cells [7], we asked whether RIP1 is also involved in chemosensitization by BV6. To address this question, we used both genetic and pharmacological approaches. Genetically, RIP1 was transiently knocked down by siRNA oligonucleotides using two different siRNA oligonucleotides and knockdown efficacy was controlled by Western blotting (Figure 4A). Interestingly, we found that silencing of RIP1 significantly reduced DOX/BV6-induced apoptosis, while it did not rescue VCR/BV6-mediated apoptosis (Figure 4B). These results were independently confirmed by stable knockdown of RIP1 using a short-hairpin RNA (shRNA) vector (suppl. Figure 3A, 3B). Also, the pharmacological RIP1 kinase inhibitor necrostatin-1 (Nec-1) significantly decreased DOX/BV6- but not VCR/BV6-mediated apoptosis (Figure 4C). Together, this set of experiments shows that RIP1 is required for DOX/BV6-induced apoptosis, while it is largely dispensable for VCR/BV6-mediated apoptosis.

### VCR/BV6 cotreatment engages the mitochondrial pathway

**Figure 5:**
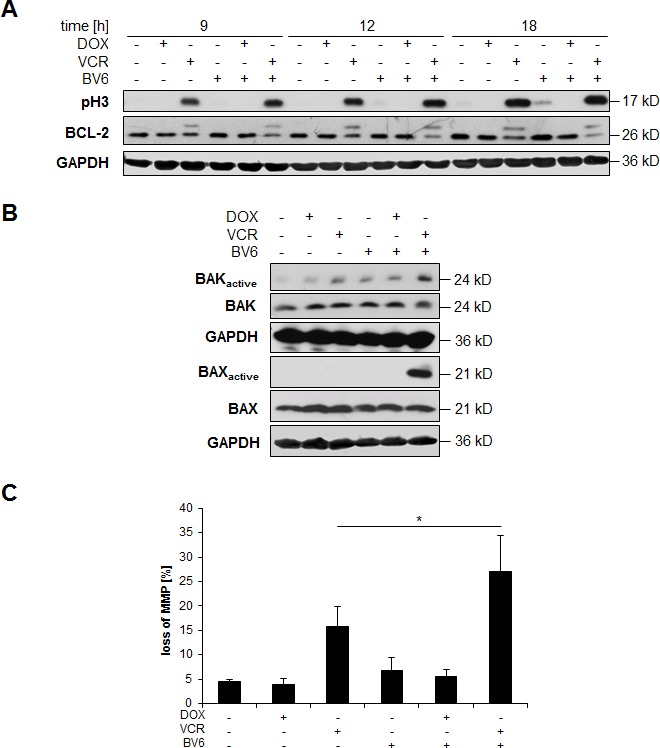
VCR/BV6 cotreatment engages the mitochondrial pathway **A.** SH-EP cells were treated with 5 μM BV6 and/or 0.05 μg/ml DOX and/or 5 nM VCR for indicated times and expression of BCL-2 or phosphorylated histone H3 (pH3) was analyzed by Western blotting; expression of GAPDH served as loading control. **B.** SH-EP cells were treated with 5 μM BV6 and/or 0.05 μg/ml DOX and/or 5 nM VCR for 18 hours. BAK or BAX were immunoprecipitated using active conformation-specific antibodies and expression of active and total BAK or BAX was analyzed by Western blotting, GAPDH served as loading control. **C.** SH-EP cells were treated with 5 μM BV6 and/or 0.05 μg/ml DOX and/or 5 nM VCR for 18 hours. MMP was analyzed by flow cytometry using JC-1 staining. Data are shown as mean and SD of three independent experiments performed in triplicate; *, *P* < 0.05.

BCL-2 has been described to become inactivated via phosphorylation during mitotic arrest caused for example by treatment with microtubule-interfering drugs such as VCR [14]. Since proteins of the BCL-2 family play an important role in the control of mitochondrial outer membrane permeabilization, we hypothesized that the mitochondrial pathway of apoptosis is preferentially engaged during VCR/BV6-mediated apoptosis. To test this hypothesis, we analyzed the phosphorylation status of BCL-2 by Western blotting, as the phosphorylated BCL-2 migrates slower than the unphosphorylated form and can be detected as a separate band on SDS-PAGE. Indeed, treatment with VCR alone and in combination with BV6 resulted in the appearance of an additional higher band of BCL-2 (Figure 5A), consistent with increased phosphorylation of BCL-2. Addition of λ-phosphatase blocked this bandshift in BCL-2, underscoring that it is caused by phosphorylation (suppl. Figure 4). In parallel, we detected increased phosphorylation of histone H3, a specific marker of mitosis [15] in VCR-treated and VCR/BV6-cotreated cells (Figure 5A). These findings indicate that VCR promotes inactivation of BCL-2 via increased phosphorylation during mitotic arrest.

To investigate whether BCL-2 inactivation by phosphorylation leads to activation of the proapoptotic multidomain proteins BAX and BAK, we analyzed the activation status of BAX and BAK by immunoprecipitation. Upon activation, BAX and BAK undergo a conformational change that can be detected by active conformation-specific antibodies. Interestingly, we found increased levels of the activated forms of BAX and BAK in particular upon VCR/BV6 cotreatment (Figure 5B). To test whether activation of BAX and BAK leads to mitochondrial outer membrane permeabilization, we analyzed MMP. VCR and BV6 cooperated to trigger loss of mitochondrial membrane potential compared to treatment with VCR alone (Figure 5C).

### BCL-2 overexpression rescues in particular VCR/BV6-induced apoptosis

**Figure 6:**
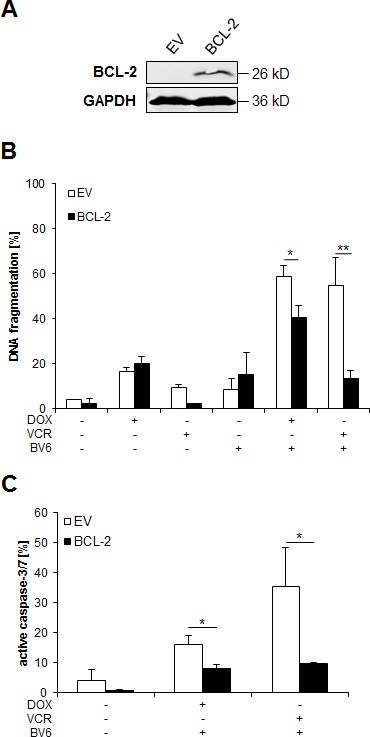
BCL-2 overexpression rescues in particular VCR/BV6-induced apoptosis **A** and **B.** SH-EP cells were stably transduced with empty vector of murine BCL-2 and expression of murine BCL-2 was analyzed by Western blotting; expression of GAPDH served as loading control (A). SH-EP cells were treated with either 5 μM BV6 and/or 0.05 μg/ml DOX or 5 μM BV6 and/or 5 nM VCR for 72 hours (B). Apoptosis was determined by analysis of DNA fragmentation of PI-stained nuclei using flow cytometry. Data are shown as mean and SD of three independent experiments performed in triplicate; *, *P* < 0.05; **, *P* < 0.01. **C.** Empty vector (EV) or BCL-2 overexpressing SH-EP cells were treated with 5 μM BV6 and 0.05 μg/ml DOX or 5 μM BV6 and 5 nM VCR for 48 hours. Caspase activity was determined by Cell Event Caspase-3/7 Green Detection Reagent and ImageXpress Micro XLS system. Data are shown as mean and SD of three independent experiments performed in triplicate; *, *P* < 0.05.

To further explore the functional relevance of BCL-2 and the mitochondrial pathway of apoptosis, we overexpressed BCL-2. Ectopic expression of BCL-2 was confirmed by Western blotting (Figure 6A). Of note, BCL-2 overexpression almost completely suppressed VCR/BV6-mediated apoptosis and decreased the amount of cells with active caspase-3/7, while it partially decreased DOX/BV6-induced apoptosis and caspase-3/7 activation (Figure 6B, 6C). This indicates that the mitochondrial pathway of apoptosis is particularly important for mediating VCR/BV6-induced apoptosis.

## DISCUSSION

In the present study, we investigated the ability of Smac mimetics to sensitize NB cells to chemotherapy as well as the underlying molecular mechanisms of action. Here, we report that Smac mimetics prime NB cells for apoptosis upon treatment with chemotherapeutic drugs including the topoisomerase II inhibitor DOX and microtubule-interfering drugs such as VCR, VBL and VNR. BV6 acts together with DOX or VCR not only to reduce cell viability and to induce DNA fragmentation, but also to suppress long-term clonogenic survival, demonstrating the efficacy of these combinations.

Interestingly, our mechanistic studies identify distinct initial signaling events during apoptotic cell death depending on the chemotherapeutic drug that is used in combination with BV6. While RIP1 is required for DOX/BV6-induced apoptosis, RIP1 turns out to be largely dispensable for VCR/BV6-mediated cell death. This conclusion is supported by both genetic and pharmacological inhibition of RIP1 showing that stable or transient knockdown of RIP1 by RNAi as well as pharmacological inhibition of RIP1 by Nec-1 significantly rescue DOX/BV6-, but not VCR/BV6-induced apoptosis. By comparison, engagement of the mitochondrial pathway of apoptosis is a critical event in particular during VCR/BV6-mediated apoptosis. Accordingly, treatment with VCR or VCR/BV6 causes phosphorylation and thus inactivation of BCL-2 during VCR-imposed mitotic arrest, leading to activation of the proapoptotic multidomain proteins BAX and BAK and loss of MMP. Also, caspase-9, the initiator caspase in the mitochondrial pathway of apoptosis, was predominately activated by VCR/BV6 cotreatment. The crucial role of mitochondrial signaling is underlined by rescue experiments showing that overexpression of BCL-2 almost completely blocks VCR/BV6-induced apoptosis, whereas BCL-2 overexpression partially decreases DOX/BV6-induced apoptosis. Subsequent to these distinct initial signaling events, both DOX/BV6 and VCR/BV6 combination treatments share a common effector phase of apoptosis, which relies on activation of caspases and caspase-dependent cell death, as the broad-range caspase inhibitor zVAD.fmk markedly reduces DOX/BV6- as well as VCR/BV6-induced apoptosis. Since Smac mimetics such as BV6 antagonize XIAP in addition to cIAP1 and cIAP2 proteins, the Smac mimetic-induced release of XIAP-mediated inhibition of caspase-9, -7 and -3 results in enforced caspase activation.

While the Smac mimetic LBW242 has been described in the past to enhance the efficacy of chemotherapy against NB *in vitro* and *in vivo* [9], the underlying mechanisms remained elusive. Thus, the novelty of our current study resides in the demonstration that BV6-mediated chemosensitization of NB cells is mediated via distinct initial signaling events in a chemotherapeutic drug-dependent manner. We identify a crucial role of RIP1 for DOX/BV6-induced apoptosis, whereas apoptosis upon VCR/BV6 cotreatment critically depends on the engagement of the mitochondrial pathway of apoptosis.

RIP1 has previously been reported to constitute a central signaling hub for Smac mimetic-mediated sensitization of cancer cells to anticancer drugs. In acute lymphoblastic leukemia (ALL), we identified RIP1 as a critical regulator of the synergism of Smac mimetics and Cytarabine, which mediates the formation of a RIP1/caspase-8/FADD complex via an autocrine/paracrine loop of tumor necrosis factor-α (TNFα) and is necessary for activation of caspase-8 and -3, mitochondrial perturbations and apoptosis [16]. In addition, we demonstrated that BV6 primes glioblastoma cells in a RIP1-dependent manner for Temozolomide (TMZ), the first-line chemotherapeutic agent in the treatment of glioblastoma, since knockdown of RIP1 significantly reduced BV6- and TMZ-induced caspase-8 activation and apoptosis [17]. Besides combination studies with chemotherapeutic drugs, RIP1 has been shown to be required for Smac mimetic-based sensitization to other cytotoxic stimuli. For example, RIP1 was reported to be required for apoptosis induced by Smac mimetics together with agonistic monoclonal antibodies against TRAIL receptors in NB [7], glioblastoma [18] and rhabdomyosarcoma cells [19] as well as for the synergistic interaction of Smac mimetics together with glucocorticoids in ALL [20].

While an autocrine/paracrine TNFα loop has been demonstrated to mediate cell death in response to Smac mimetics alone and in combination with chemotherapeutic drugs in a variety of cancers [12, 16, 21–24], in our present study TNFα turned out to be dispensable for both DOX/BV6- and VCR/BV6-mediated apoptosis. Similarly, sensitization for chemotherapy by Smac mimetics has been described to occur in a TNFα-independent fashion [9, 17, 25]. This points to both TNFα-dependent and TNFα-independent signaling pathways during Smac mimetic/chemotherapy-triggered cell death.

In contrast to these studies showing the requirement of RIP1 for Smac mimetic-mediated sensitization to various cytotoxic stimuli, RIP1 was also demonstrated to be dispensable for Smac mimetic-based combination therapies [25, 26]. Interestingly, Greer *et al.* reported that silencing of caspase-9, but not RIP1 rescued non-small cell lung cancers cells from cell death upon combined treatment with the Smac mimetic JP1201 and the vinca alkaloid VNR [25]. As caspase-9 represents the initiator caspase of the intrinsic apoptotic pathway, these data support our conclusion that the mitochondrial pathway of apoptosis plays a critical role in mediating cell death in combination treatment with Smac mimetic together with VCR as one of the vinca alkaloids. However, the impact of the mitochondrial apoptotic pathway likely depends on the context including the cytotoxic stimulus, since we previously reported that XIAP inhibition combined with the death receptor ligand TRAIL can bypass BCL-2-imposed resistance of pancreatic carcinoma cells by switching type II cells, which depend on the mitochondrial contribution as an amplification step to the death receptor pathway, into type I cells in which TRAIL-induced caspase activation and apoptosis proceeds irrespectively of high BCL-2 levels [27].

The present study has important implications, as it emphasizes the potential of combination regimens with Smac mimetics and commonly used anticancer drugs to increase chemosensitivity of NB. Smac mimetic-based combination therapies might also reduce the risk of toxic side effects, as lower concentrations of chemotherapeutics might be sufficient in combination regimens due to the cooperative effects. This might be particularly relevant for drugs such as DOX and VCR for which drug toxicity such as cardiotoxicity and neurotoxicity can cause serious concerns [28, 29]. Since DOX and vinca alkaloids form part of standard chemotherapy in NB [1] and since Smac mimetics are under evaluation in early clinical trials [30], our study provides promising approaches for the development of novel experimental treatment protocols.

## MATERIALS AND METHODS

### Cell culture and chemicals

NB cell lines were obtained from American Type Culture Collection (ATCC) (Manassas, VA, USA) and maintained in DMEM GlutaMAX^™^-l or RPMI medium (Life Technologies, Inc., Darmstadt, Germany), supplemented with 10% fetal calf serum (FCS), 1% penicillin/streptomycin, 1 mM sodium pyruvate and 10 mM HEPES (all from Life Technologies, Inc.). LAN-5 cells were grown in flasks coated with rat tail I collagen (BD Biosciences, San Jose, CA, USA). Doxorubicin, VCR, VBL and VNR were purchased from Sigma (Deisenhofen, Germany); zVAD.fmk from Bachem (Heidelberg, Germany), Enbrel from Pfizer (Berlin, Germany), Nec-1 from Biomol (Biomol GmbH, Hamburg, Germany) and TNFα from Biochrom (Biochrom GmbH, Berlin, Germany). The bivalent Smac mimetic BV6 was kindly provided by Genentech (South San Francisco, CA, USA), chemicals were purchased from Sigma unless otherwise indicated.

### Determination of apoptosis, cell viability, colony formation and caspase-3/7 activity

Apoptosis was determined by flow cytometric analysis (FACSCanto II, BD Biosciences, Heidelberg, Germany) of DNA fragmentation of propidium iodide (PI)-stained nuclei as described previously [31]. Cell viability was assessed by crystal violet assay using crystal violet solution (0.5% crystal violet, 30% ethanol, 3% formaldehyde). Plates were then rinsed with water and crystal violet incorporated by the cells was re-solubilized in a solution containing 1% SDS. Absorbance at 550 nm was measured using a microplate reader (Infinite M100, Tecan, Crailsheim, Germany). Results are expressed as percentage of untreated controls. For colony formation assay, 100 cells per well were seeded in 6-well plates after treatment with either 5 μM BV6 for 11 hours and/or 0.05 μg/ml DOX for 1 hour or 5 μM BV6 and/or 5 nM VCR for 11 hours. Cells were cultured in drug-free medium for additional 13 days before fixation and staining with 0.5% crystal violet, 30% ethanol and 3% formaldehyde. Colonies were counted macroscopically. Caspase-3/7 activity was detected by Cell Event Caspase-3/7 Green Detection Reagent (Life Technologies, Inc.) and ImageXpress Micro XLS system (Molecular Devices, Biberach an der Riss, Germany).

### Overexpression and RNA interference

Stable overexpression of dominant-negative IκBα-SR was performed by retroviral vectors. Shortly, PT67 cells were transfected with 4 μg of pCFG5-IEGZ plasmid (empty vector; IκBα (S32A/S36A)) supplied with Lipofectamine 2000 (Life Technologies, Inc.). Virus-containing supernatant was collected, sterile-filtered, and used for spin transduction at 37°C in the presence of 8 μg/ml polybrene. For selection, 1 μg/ml zeocin (Invitrogen) was used. For transient knockdown of RIP1, cells were reversely transfected with 5 pmol Silencer Select (Life Technologies, Inc.) control siRNA (4390844) or targeting siRNA (s16651 and s16653 for RIP1), respectively, using Lipofectamine RNAiMAX reagent (Life Technologies, Inc.) and Opti-MEM medium (Life Technologies, Inc.). For stable knockdown of RIP1, cells were transfected with lentiviral shRNA plasmids targeting RIP1 sequence (5′-cactagtctgacggataa-3′) or a control sequence with no corresponding part in the human genome (5′-atcatgtagatacgctca-3′). For selection, 1 μg/ml puromycin (Invitrogen) was used.

Murine BCL-2 was stably overexpressed by using lentiviral vectors. Shortly, Phoenix cells were transfected with 20 μg of pMSCV plasmid (empty vector, BCL-2) using calcium phosphate transfection. Virus-containing supernatant was collected, sterile-filtered, and used for spin transduction at 37°C in the presence of 8 μg/ml polybrene. Transduced SH-EP were selected with 2 μg/ml blasticidin (Invitrogen).

### Western blot analysis

Western blot analysis was performed as described previously [31], using the following antibodies: cIAP1, α-Tubulin, pH3, RIP1, BCL-2 (BD, New Jersey, USA), p-IκBα, IIκBα, caspase-3, caspase-9 (Cell Signaling, Beverly, MA), caspase-8 (Enzo Life Science, Lörrach, Germany), GAPDH (HyTest, Turku, Finland), BCL-2 (Life Technologies, Inc.). Goat anti-mouse IgG, goat anti-rabbit IgG and donkey anti-goat IgG conjugated to horseradish peroxidase (Santa Cruz Biotechnology) were used as secondary antibodies for enhanced chemiluminescence (Amersham Bioscience, Freiburg, Germany) and Donkey anti-mouse IgG, donkey anti-rabbit IgG or donkey anti-goat IgG labeled with IRDye infrared dyes were used for fluorescence detection (Odyssey Imaging System, LI-COR Bioscience, Bad Homburg, Germany). Representative blots of at least two independent experiments are shown.

### Determination of BAX/BAK activation and MMP

BAX and BAK activation was determined by immunoprecipitation of active conformation by specific antibodies as previously described [32]. Briefly, cells were lysed in CHAPS buffer (1% CHAPS, 150 mM NaCl, 10 mM HEPES pH 7.4). 1 mg protein was incubated overnight at 4°C with 2 g mouse monoclonal anti-BAX antibody (clone 6A7; Sigma) or 0.5 μg mouse monoclonal anti-BAK antibody (AB-1, clone TC-100; Merck, Darmstadt, Germany) and 10 μl pan-mouse IgG Dynabeads (Dako, Hamburg, Germany), washed with CHAPS buffer, and analyzed by Western blotting using rabbit polyclonal anti-BAX NT (Merck) antibody or rabbit polyclonal anti-BAK antibody (BD Biosciences). MMP was analyzed by flow cytometry using JC-1 dye (Life Technologies, Inc.).

### Statistical analysis

Statistical significance, when comparing two groups, was assessed by Student's t-Test (two-tailed distribution, two-samples with equal variance) using Microsoft Excel (Microsoft Deutschland GmbH, UnterschleiΔheim, Germany); *, *P* < 0.05; **, *P* < 0.01; ***, *P* < 0.001. Drug interactions were analyzed by the CI method based on that described by Chou [33] using CalcuSyn software (Biosoft, Cambridge, UK). CI < 0.9 indicates synergism, 0.9 - 1.1 additivity and > 1.1 antagonism.

## SUPPLEMENTARY MATERIAL TABLE AND FIGURES



## References

[1] Maris JM, Hogarty MD, Bagatell R, Cohn SL (2007). Neuroblastoma. Lancet.

[2] Fulda S, Debatin KM (2006). Extrinsic versus intrinsic apoptosis pathways in anticancer chemotherapy. Oncogene.

[3] Fulda S (2009). Tumor resistance to apoptosis. Int J Cancer.

[4] Ashkenazi A (2008). Directing cancer cells to self-destruct with pro-apoptotic receptor agonists. Nat Rev Drug Discov.

[5] Fulda S, Galluzzi L, Kroemer G (2010). Targeting mitochondria for cancer therapy. Nat Rev Drug Discov.

[6] Fulda S, Vucic D (2012). Targeting IAP proteins for therapeutic intervention in cancer. Nat Rev Drug Discov.

[7] Abhari BA, Cristofanon S, Kappler R, von Schweinitz D, Humphreys R, Fulda S (2013). RIP1 is required for IAP inhibitor-mediated sensitization for TRAIL-induced apoptosis via a RIP1/FADD/caspase-8 cell death complex. Oncogene.

[8] Giagkousiklidis S, Vogler M, Westhoff MA, Kasperczyk H, Debatin KM, Fulda S (2005). Sensitization for gamma-irradiation-induced apoptosis by second mitochondria-derived activator of caspase. Cancer Res.

[9] Eschenburg G, Eggert A, Schramm A, Lode HN, Hundsdoerfer P (2012). Smac mimetic LBW242 sensitizes XIAP-overexpressing neuroblastoma cells for TNF-alpha-independent apoptosis. Cancer Res.

[10] Vogler M, Giagkousiklidis S, Genze F, Gschwend JE, Debatin KM, Fulda S (2005). Inhibition of clonogenic tumor growth: a novel function of Smac contributing to its antitumor activity. Oncogene.

[11] Rapino F, Naumann I, Fulda S (2013). Bortezomib antagonizes microtubule-interfering drug-induced apoptosis by inhibiting G2/M transition and MCL-1 degradation. Cell Death Dis.

[12] Varfolomeev E, Blankenship JW, Wayson SM, Fedorova AV, Kayagaki N, Garg P, Zobel K, Dynek JN, Elliott LO, Wallweber HJ, Flygare JA, Fairbrother WJ, Deshayes K (2007). IAP antagonists induce autoubiquitination of c-IAPs, NF-kappaB activation, and TNFalpha-dependent apoptosis. Cell.

[13] Eckhardt I, Roesler S, Fulda S (2013). Identification of DR5 as a critical, NF-kappaB-regulated mediator of Smac-induced apoptosis. Cell Death Dis.

[14] Haldar S, Basu A, Croce CM (1997). Bcl2 is the guardian of microtubule integrity. Cancer Res.

[15] Hans F, Dimitrov S (2001). Histone H3 phosphorylation and cell division. Oncogene.

[16] Loeder S, Fakler M, Schoeneberger H, Cristofanon S, Leibacher J, Vanlangenakker N, Bertrand MJ, Vandenabeele P, Jeremias I, Debatin KM, Fulda S (2012). RIP1 is required for IAP inhibitor-mediated sensitization of childhood acute leukemia cells to chemotherapy-induced apoptosis. Leukemia.

[17] Wagner L, Marschall V, Karl S, Cristofanon S, Zobel K, Deshayes K, Vucic D, Debatin KM, Fulda S (2013). Smac mimetic sensitizes glioblastoma cells to Temozolomide-induced apoptosis in a RIP1- and NF-kappaB-dependent manner. Oncogene.

[18] Cristofanon S, Abhari BA, Krueger M, Tchoghandjian A, Momma S, Calaminus C, Vucic D, Pichler BJ, Fulda S (2015). Identification of RIP1 as a critical mediator of Smac mimetic-mediated sensitization of glioblastoma cells for Drozitumab-induced apoptosis. Cell Death Dis.

[19] Basit F, Humphreys R, Fulda S (2012). RIP1 Protein-dependent Assembly of a Cytosolic Cell Death Complex Is Required for Inhibitor of Apoptosis (IAP) Inhibitor-mediated Sensitization to Lexatumumab-induced Apoptosis. J Biol Chem.

[20] Belz K, Schoeneberger H, Wehner S, Weigert A, Bonig H, Klingebiel T, Fichtner I, Fulda S (2014). Smac mimetic and glucocorticoids synergize to induce apoptosis in childhood ALL by promoting ripoptosome assembly. Blood.

[21] Vince JE, Wong WW, Khan N, Feltham R, Chau D, Ahmed AU, Benetatos CA, Chunduru SK, Condon SM, McKinlay M, Brink R, Leverkus M, Tergaonkar V (2007). IAP antagonists target cIAP1 to induce TNFalpha-dependent apoptosis. Cell.

[22] Petersen SL, Wang L, Yalcin-Chin A, Li L, Peyton M, Minna J, Harran P, Wang X (2007). Autocrine TNFalpha signaling renders human cancer cells susceptible to Smac-mimetic-induced apoptosis. Cancer Cell.

[23] Probst BL, Liu L, Ramesh V, Li L, Sun H, Minna JD, Wang L (2010). Smac mimetics increase cancer cell response to chemotherapeutics in a TNF-alpha-dependent manner. Cell Death Differ.

[24] Stadel D, Cristofanon S, Abhari BA, Deshayes K, Zobel K, Vucic D, Debatin KM, Fulda S (2011). Requirement of nuclear factor kappaB for Smac mimetic-mediated sensitization of pancreatic carcinoma cells for gemcitabine-induced apoptosis. Neoplasia.

[25] Greer RM, Peyton M, Larsen JE, Girard L, Xie Y, Gazdar AF, Harran P, Wang L, Brekken RA, Wang X, Minna JD (2011). SMAC Mimetic (JP1201) Sensitizes Non-Small Cell Lung Cancers to Multiple Chemotherapy Agents in an IAP-Dependent but TNF-alpha-Independent Manner. Cancer Res.

[26] Liese J, Abhari BA, Fulda S (2015). Smac mimetic and oleanolic acid synergize to induce cell death in human hepatocellular carcinoma cells. Cancer Lett.

[27] Vogler M, Walczak H, Stadel D, Haas TL, Genze F, Jovanovic M, Gschwend JE, Simmet T, Debatin KM, Fulda S (2008). Targeting XIAP bypasses Bcl-2-mediated resistance to TRAIL and cooperates with TRAIL to suppress pancreatic cancer growth *in vitro* and *in vivo*. Cancer Res.

[28] Quiles JL, Huertas JR, Battino M, Mataix J, Ramírez-Tortosa MC (2002). Antioxidant nutrients and adriamycin toxicity. Toxicology.

[29] Magge RS, DeAngelis LM (2015). The double-edged sword: Neurotoxicity of chemotherapy. Blood Rev.

[30] Fulda S (2014). Molecular pathways: targeting death receptors and smac mimetics. Clin Cancer Res.

[31] Fulda S, Sieverts H, Friesen C, Herr I, Debatin KM (1997). The CD95 (APO-1/Fas) system mediates drug-induced apoptosis in neuroblastoma cells. Cancer Res.

[32] Hacker S, Dittrich A, Mohr A, Schweitzer T, Rutkowski S, Krauss J, Debatin KM, Fulda S (2009). Histone deacetylase inhibitors cooperate with IFN-gamma to restore caspase-8 expression and overcome TRAIL resistance in cancers with silencing of caspase-8. Oncogene.

[33] Chou TC, Chou TC (1991). The median-effect principle and the combination index for quantitation of synergism and antagonism. Synergism and antagonism in chemotherapy.

