# Supplementary material for: Differential role of RIP1 in Smac mimetic-mediated chemosensitization of neuroblastoma cells
Source: Oncotarget. 2015 Nov 12;6(39):41522–34. doi: 10.18632/oncotarget.6308 (PMC4747171; doi:10.18632/oncotarget.6308)
Supplement: Supplementary file 1 [file oncotarget-06-41522-s001.pdf]

Differential role of RIP1 in Smac mimetic-mediated chemosensitization of neuroblastoma cells

Supplementary Material

Supplemental Materials and Methods

IAP inhibitor 3 was described by Chao *et al.*[1] and kindly provided by Idun Pharmaceuticals now Pfizer (Groton, CT, USA). PBLs were isolated from healthy donors by Ficoll separation as described in the manufacturer's protocol (Ficoll type 400 with Paque, Sigma-Aldrich, Taufkirchen, Germany), 2 x 10^5 cells/well were seeded in 96-well plates and immediately treated. VCR-resistant RD rhabdomyosarcoma cells were generated by culture in increasing concentrations of VCR until cells tolerated 10 nM VCR. To analyze the phosphorylation status of BCL-2, cell lysates were incubated with λ-phosphatase for 30 min at 30 °C.

Reference

[1]Chao B, Deckwerth TL, Furth PS, Linton SD, Spada AP, Ullman BR et al. inventors; Tetrapeptide analogs. United States patent PCT/US2005/024700 2006; 16: 02.

A

BV6 \ VCR	2.5 nM	5 nM	7.5 nM	10 nM
3 µM	0.077	0.298	0.511	0.644
5 µM	0.158	0.484	0.632	0.698

B

BV6 \ DOX	0.01 µg/ml	0.025 µg/ml	0.05 µg/ml	0.1 µg/ml
3 µM	0.606	1.054	0.931	0.190
5 µM	0.599	0.709	0.652	0.204

Suppl. Table 1: Synergistic induction of apoptosis by BV6 and VCR or DOX. Combination indices (CI) were calculated as described in Materials and Methods for apoptosis induced by combined treatment for 72 hours with indicated concentrations of BV6 and VCR (A) or BV6 and DOX (B). CI <0.9 indicates synergism, 0.9-1.1 additivity and >1.1 antagonism.

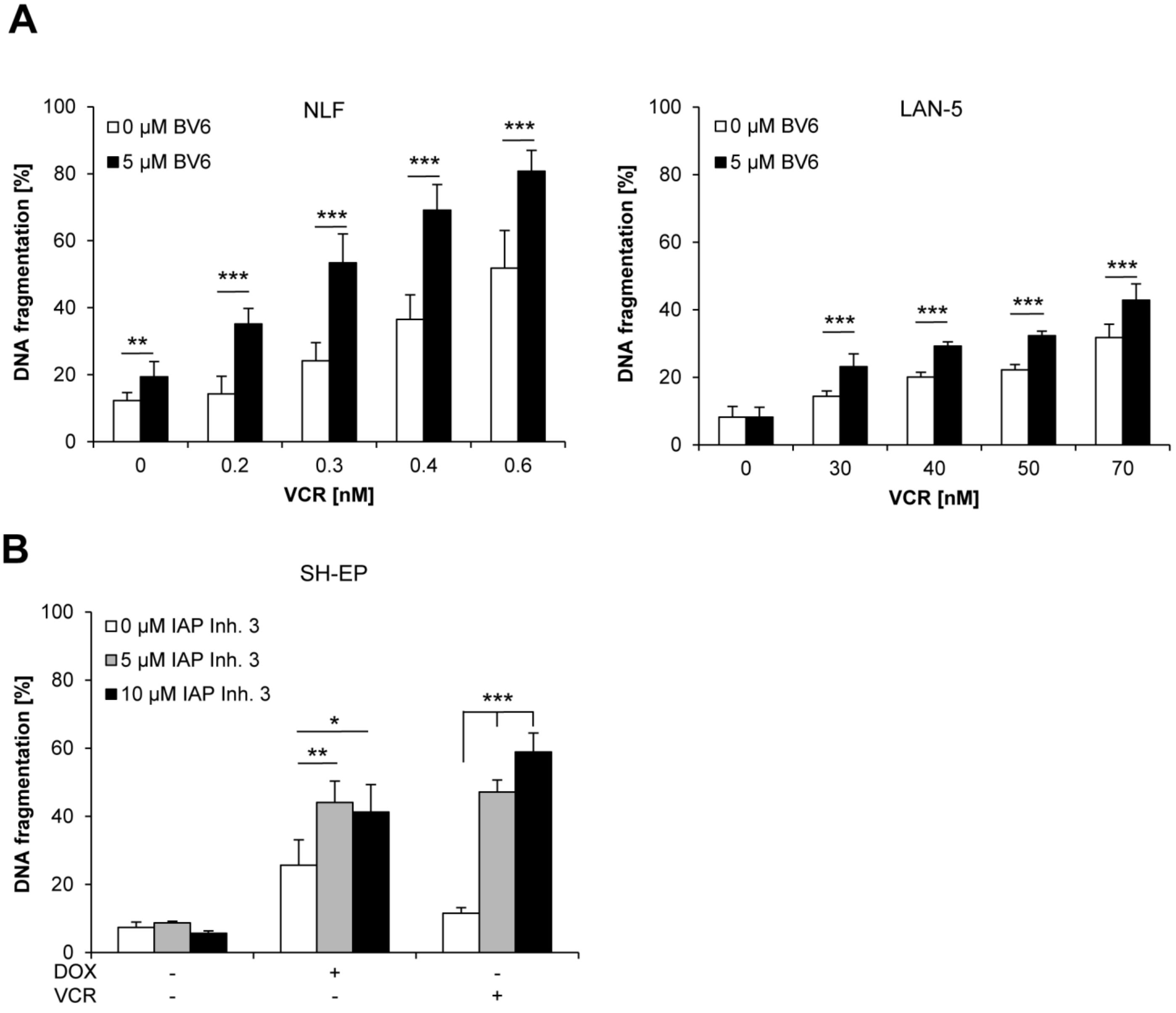
A
NLF
LAN-5
0 µM BV6
5 µM BV6
DNA fragmentation [%]
VCR [nM]
0
0.2
0.3
0.4
0.6
30
40
50
70
**
***
B
SH-EP
0 µM IAP Inh. 3
5 µM IAP Inh. 3
10 µM IAP Inh. 3
*
DOX
VCR
100
80
60
20

C

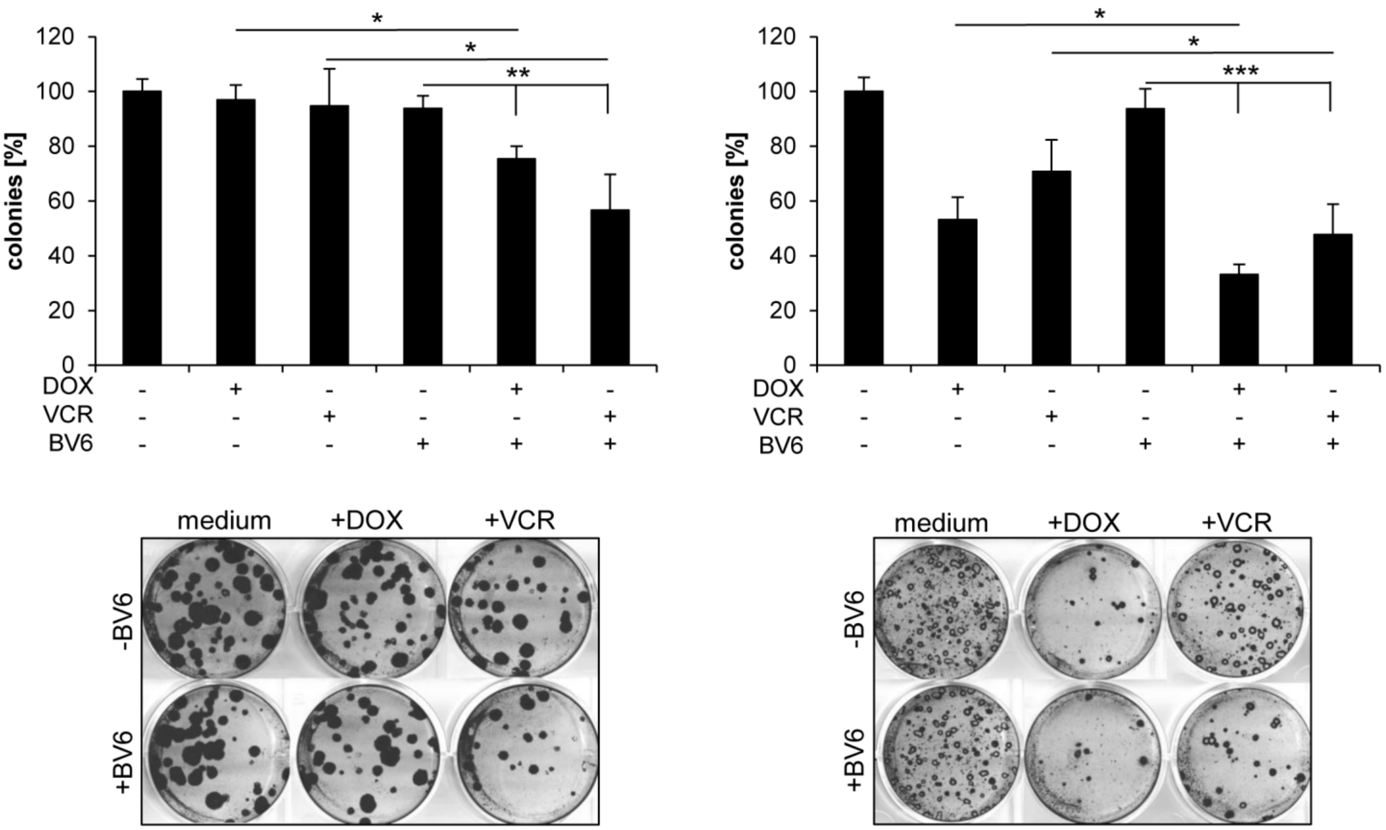
colonies [%]
0
20
40
60
80
100
120
*
*
**
***
DOX - + - - + -
VCR - - + - - +
BV6 - - - + + +
medium
+DOX
+VCR
-BV6
+BV6

D

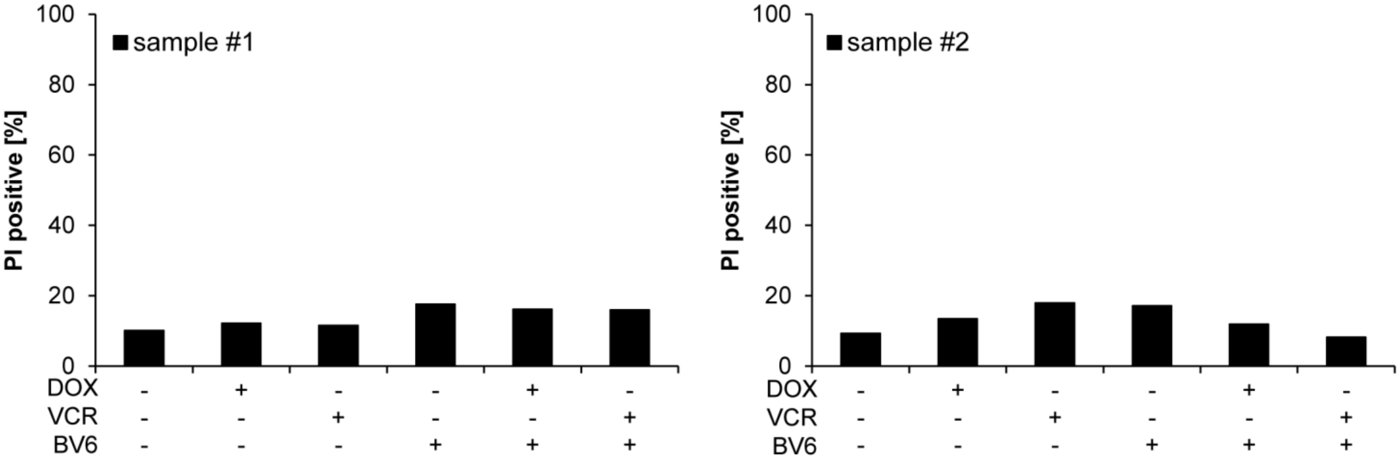
PI positive [%]
0
20
40
60
80
100
sample #1
sample #2
DOX - + - - + -
VCR - - + - - +
BV6 - - - + + +

E

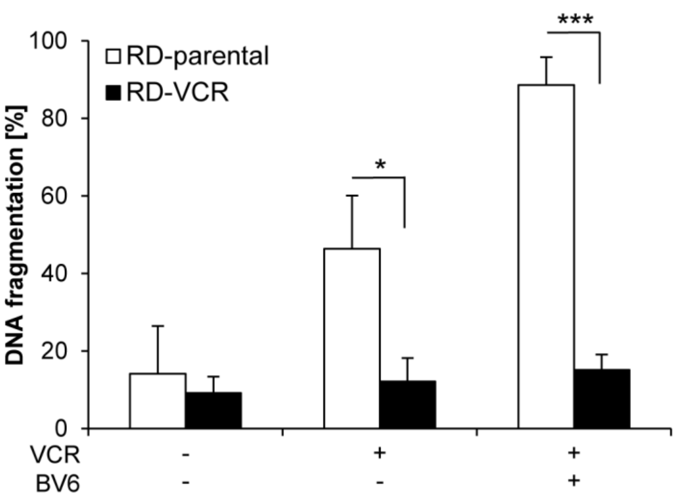

DNA fragmentation [%]
100
80
60
40
20
0
RD-parental
RD-VCR
*
***
VCR - + +
BV6 - - +

F

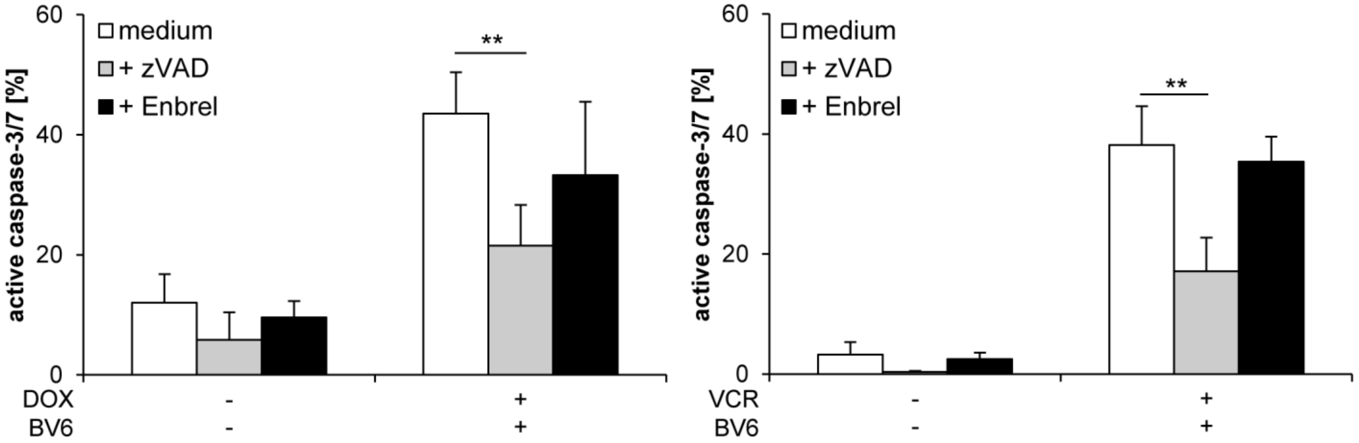

active caspase-3/7 [%]
60
40
20
0
medium
+ zVAD
+ Enbrel
**
DOX - +
BV6 - +
active caspase-3/7 [%]
60
40
20
0
medium
+ zVAD
+ Enbrel
**
VCR - +
BV6 - +

Suppl. Figure 1: Smac mimetics synergize with VCR to induce cell death in neuroblastoma cells.

A: NLF and LAN-5 cells were treated with indicated concentrations of VCR and/or 5 μM BV6 for 72 hours. Apoptosis was determined by analysis of DNA fragmentation of PI-stained nuclei using flow cytometry. Data are shown as mean +/- SD of three independent experiments performed in triplicate; **, P < 0.01; ***, P < 0.001.

B: SH-EP cells were treated with 0.05 μg/ml DOX or 5 nM VCR and indicated concentrations of IAP inhibitor 3 for 72 hours. Apoptosis was determined by analysis of DNA fragmentation of PI-stained nuclei using flow cytometry. Data are shown as mean +/- SD of three independent experiments performed in triplicate; *, P < 0.05; **, P < 0.01; ***, P < 0.001.

C: NLF cells were treated with 0.3 nM VCR and/or 5 μM BV6 and LAN-5 cells were treated with 40 nM VCR and/or 5 μM BV6 for 12 hours. Colony formation was assessed as described in Material and Methods. The number of colonies is expressed as percentage of controls (upper panels) and representative images are shown (lower panels). Data are shown as mean +/- SD of three independent experiments performed in triplicate; *, P < 0.05; **, P < 0.01; ***, P < 0.001.

D: Human PBLs from two different healthy donors (sample #1 & #2) were treated with either 5 μM BV6 and/or 0.05 μg/ml DOX or 5 μM BV6 and/or 5 nM VCR for 48 hours.

Cell death was determined by PI staining and flow cytometry. Mean values of PI-positive cells of one experiment performed in triplicate are shown.

E: RD cells were treated with 10 nM VCR and/or 10 µM BV6 for 48 hours. Apoptosis was determined by analysis of DNA fragmentation of PI-stained nuclei using flow cytometry. Data are shown as mean +/- SD of three independent experiments performed in triplicate; *, $P < 0.05$; ***, $P < 0.001$.

F: SH-EP cells were treated with 5 µM BV6 and 0.05 µg/ml DOX for 72 hours or with 5 µM BV6 and 5 nM VCR for 48 hours in the presence or absence of 20 µM zVAD.fmk or 50 µg/ml Enbrel. Caspase activity was determined by Cell Event Caspase-3/7 Green Detection Reagent and ImageXpress Micro XLS system. Data are shown as mean and SD of three independent experiments performed in triplicate; **, $P < 0.01$.

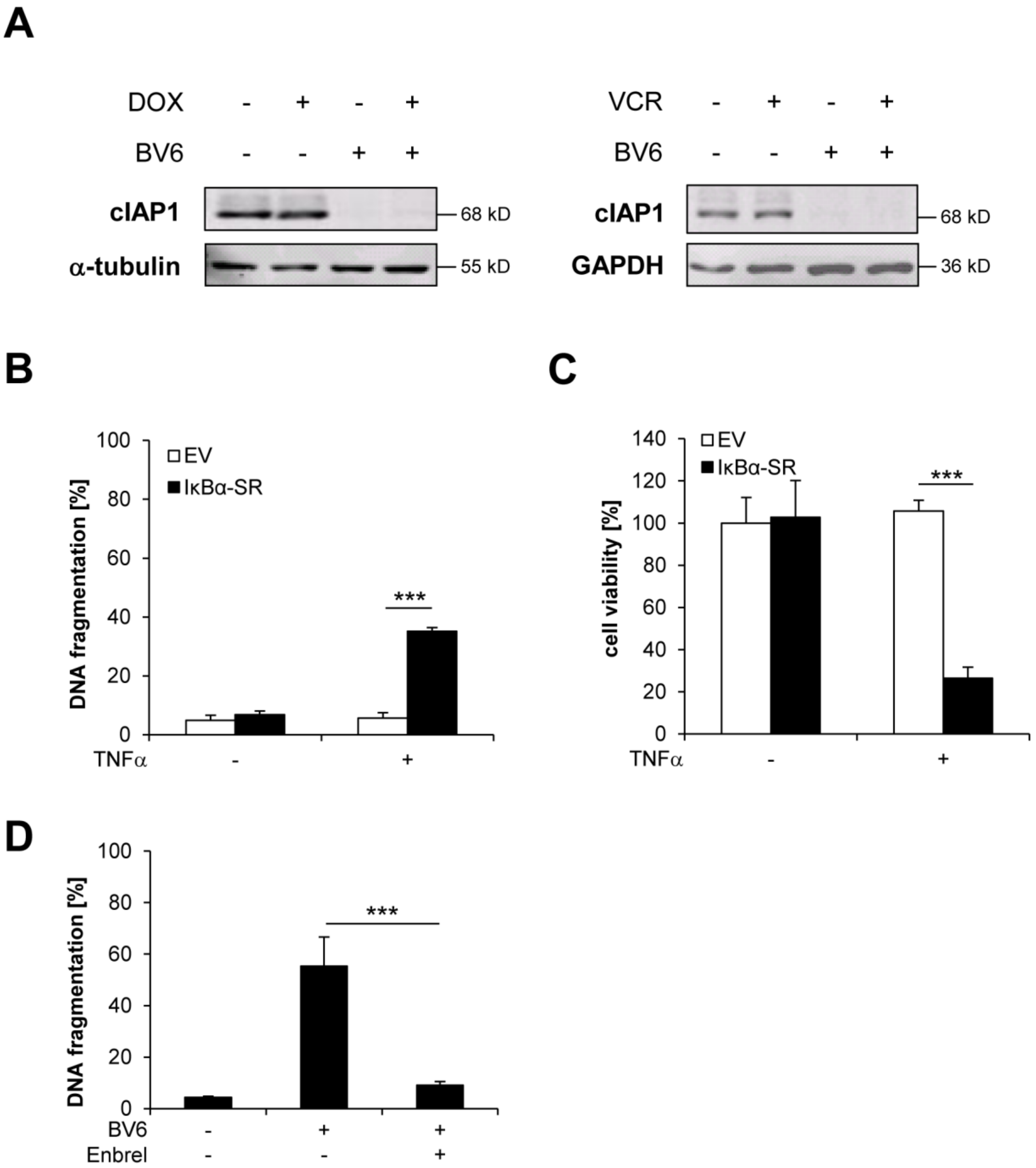
A
DOX - + - +
BV6 - - + +
cIAP1
68 kD
α-tubulin
55 kD
VCR - + - +
BV6 - - + +
cIAP1
68 kD
GAPDH
36 kD
B
DNA fragmentation [%]
100
80
60
40
20
0
EV
IκBα-SR
***
TNFα - +
C
cell viability [%]
140
120
100
80
60
40
20
0
EV
IκBα-SR
***
TNFα - +
D
DNA fragmentation [%]
100
80
60
40
20
0
***
BV6 - + +
Enbrel - - +

Suppl. Figure 2: Involvement of NF-κB in TNFα-induced cell death and TNFα in BV6-induced cell death.

A: SH-EP cells µMwereBV6µg/mltreatedand/orµMDOXwith0or.05either5 5

BV6 and/or 5 nM VCR for 1 hour and cIAP1 expression was analyzed by Western blotting; expression of α-tubulin or GAPDH served as loading controls.

B and C: SH-EP cells were stably transduced with empty vector (EV) or IκBα-SR and were treated with 10 ng/ml TNFα for 1 hour. Apoptosis was determined by analysis of DNA fragmentation of PI-stained nuclei using flow cytometry (B). Cell viability was determined by MTT assay and is expressed as the percentage of untreated controls (C). Data are shown as mean +/- SD of three independent experiments performed in triplicate; ***, P < 0.001.

D: MDA-MB-231 cells were treated with 20 nM BV6 and/or 50 µg/ml Enbrel for 72 hours. Apoptosis was determined by analysis of DNA fragmentation of PI-stained nuclei using flow cytometry. Data are shown as mean +/- SD of three independent experiments performed in triplicate; ***, P < 0.001.

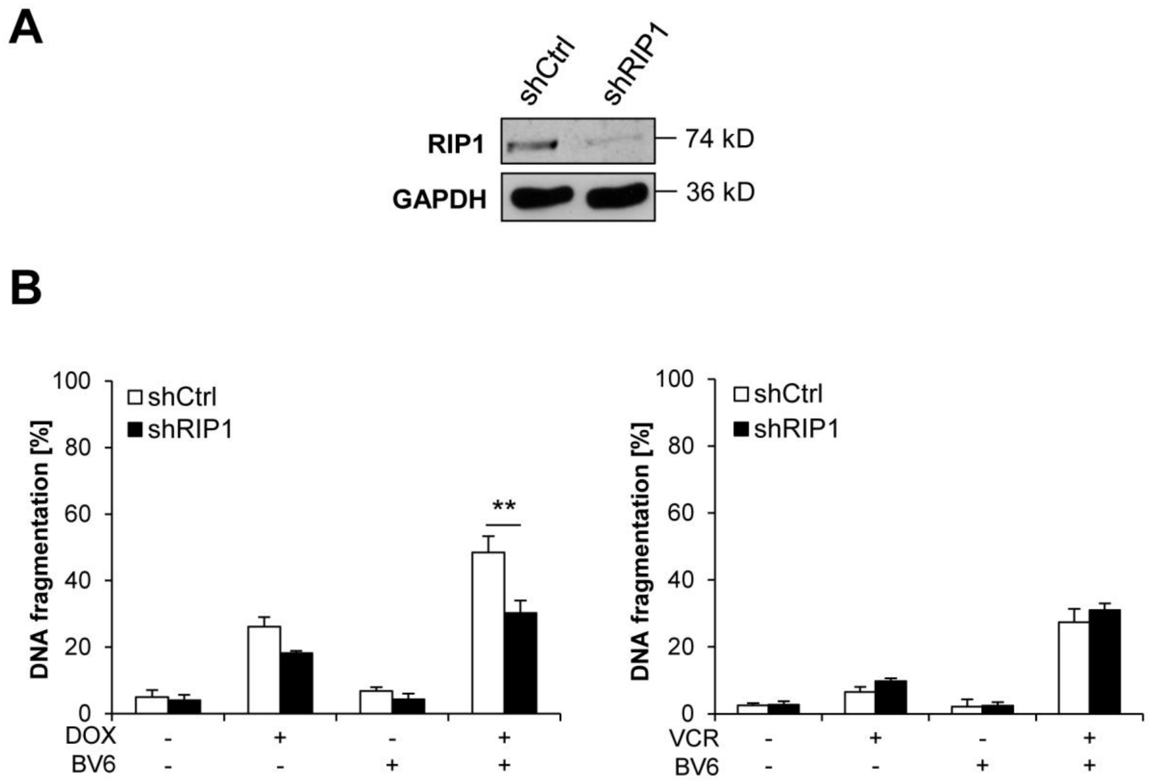

Suppl. Figure 3: RIP1 is required for DOX/BV6-induced apoptosis.

A and B: SH-EP cells were stably transfected with non-silencing shRNA (shCtrl) or a construct targeting RIP1 (shRIP1). Expression of RIP1 was analyzed by Western blotting, GAPDH served as loading control (A). SH-EP cells were treated with either 5 μM BV6 and/or 0.05 μg/ml DOX or 5 μM BV6 and/or 5 nM VCR for 72 hours (B). Apoptosis was determined by analysis of DNA fragmentation of PI-stained nuclei using flow cytometry. Data are shown as mean +/- SD of three independent experiments performed in triplicate; **, $P < 0.01$.

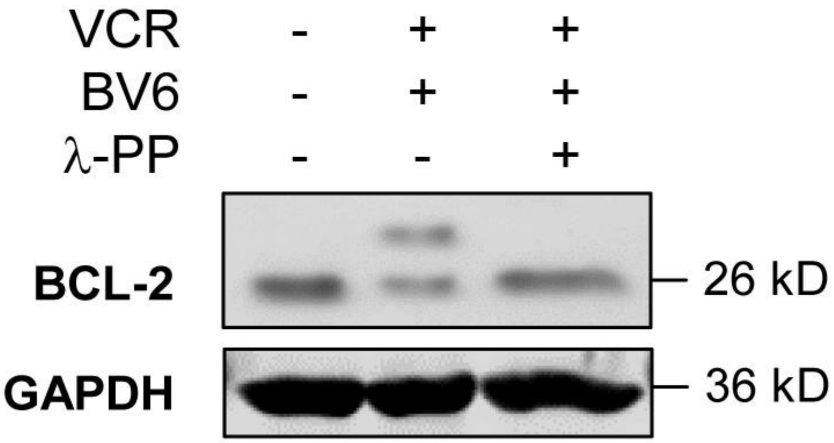

Suppl. Figure 4: VCR/BV6 co-treatment causes BCL-2 phosphorylation.

SH-EP cells were treated with 5 µM BV6 and 5 nM VCR for 18 hours. Cell lysates were incubated with λ-phosphatase (λ-PP) for 30 min at 30 °C. BCL-2 expression was analyzed by Western blotting; expression of GAPDH served as loading control.
